# Supplementary figures and images for: Human papillomavirus genomes associate with active host chromatin during persistent viral infection
Source: PLoS Pathog. 2025 Sep 2;21(9):e1013454. doi: 10.1371/journal.ppat.1013454 (PMC12440168; doi:10.1371/journal.ppat.1013454)

Figure S1

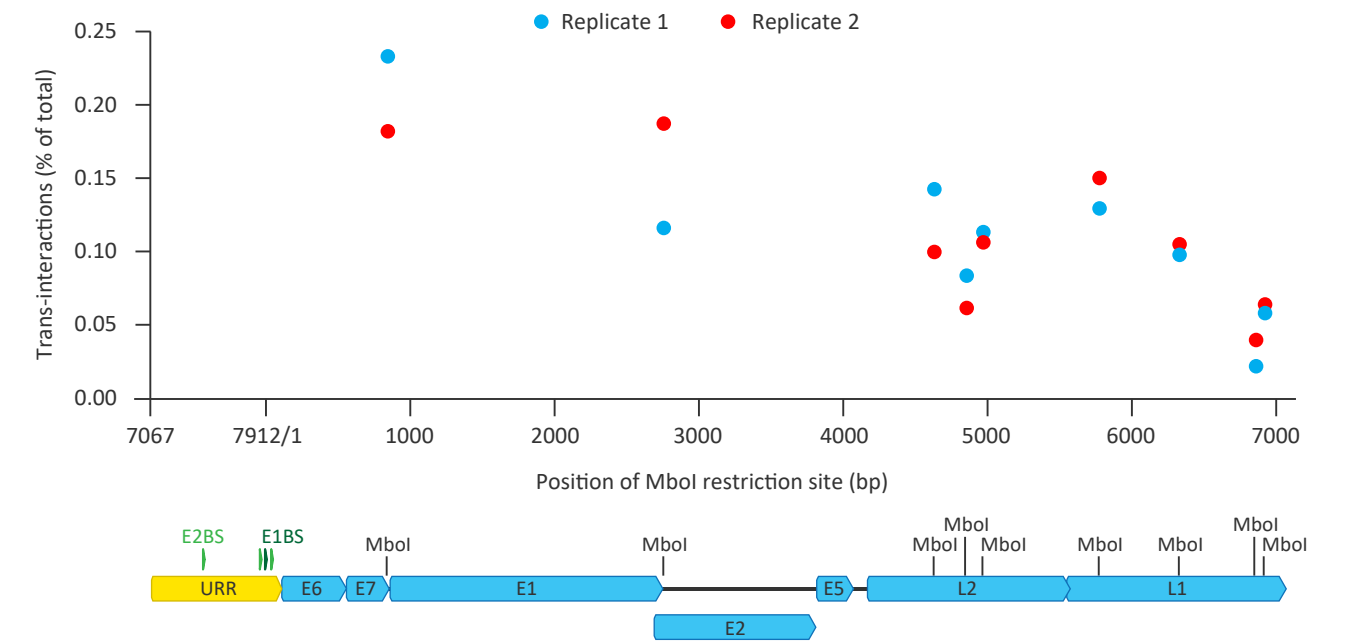

Supplement: S1 Fig — Viral-host trans-interactions mapped by HiC were aligned to the linearized HPV31 reference genome (GenBank J04353.1). The 5’ and 3’ read pairs associated with each of the indicated MboI restriction sites were plotted as the percentage of reads at each cut site relative to the total number of reads across the HPV31 genome for both HiC datasets (Replicates 1 and 2). Viral-host contacts were filtered to only include reads where the mate pair on the human genome localized to a significant 1 Mb bin listed in S1 Table. Sequencing reads that did not align to a MboI restriction site were excluded from this analysis. (PDF) [file ppat.1013454.s001.pdf]

Figure S2

A

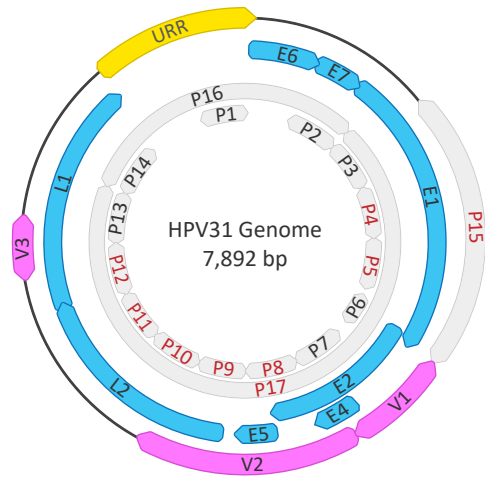

B

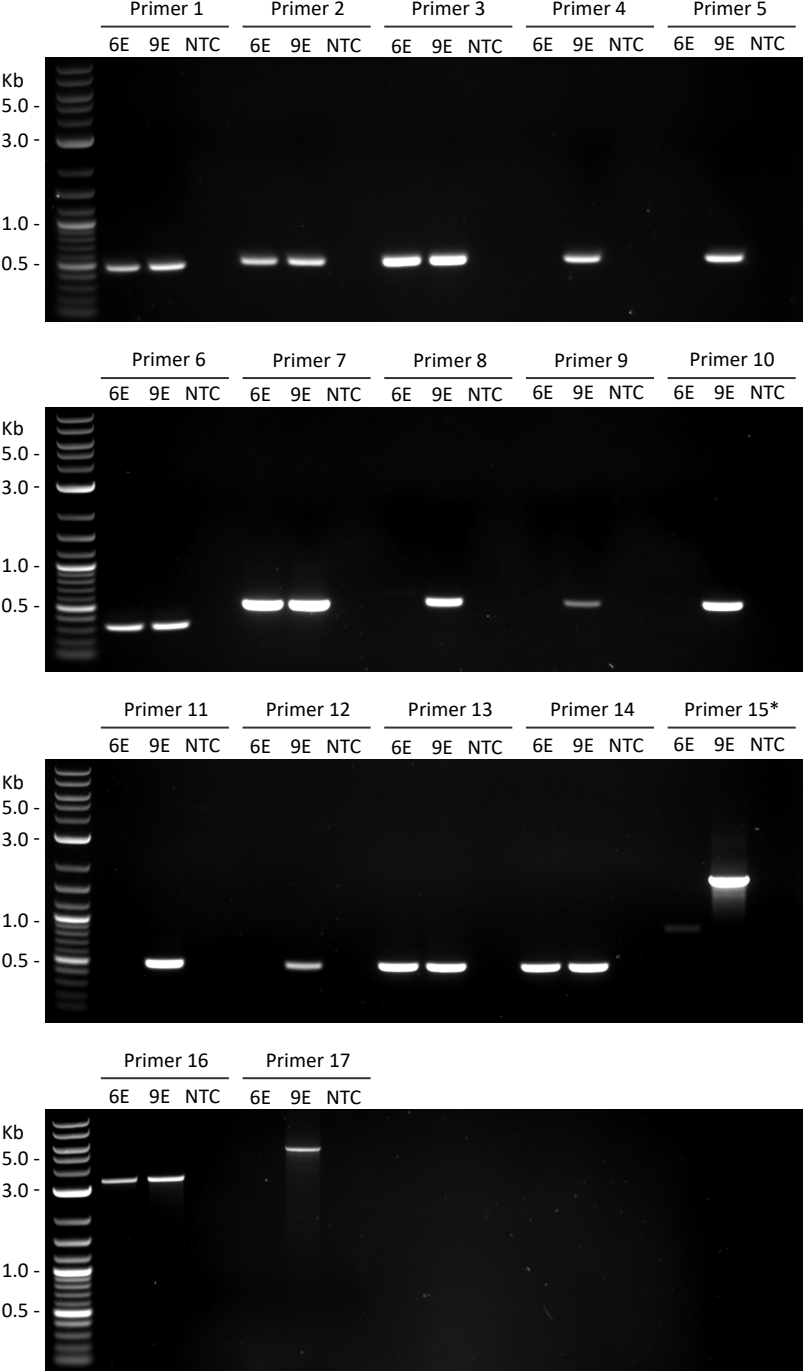

Supplement: S2 Fig — In all three 6E samples, the primary restriction site associated with the 5’ end of the trimmed sequencing reads for viewpoint 2 were not detected, and so no output files were generated. For viewpoint 1, the primary restriction site was only detected in one of the read pairs generated by paired-end sequencing in all 6E samples. This suggested that integration of the HPV31 genome had disrupted the region targeted by the 4C viewpoint 1 and 2 primers in 6E cells. To test this, gENOMIC DNA ISOLATED FROM 6E AND 9E CELLS WAS SUBJECTED TO PCR ANALYSIS USING primer pairs that span approximately every 500 bp of the HPV31 genome. A, HPV31 GENOME SHOWING THE REGIONS TARGETED BY PCR PRIMER PAIRS (GREY BARS LABELLED P1-P17 THAT CORRESPOND TO THE AMPLICONS SHOWN IN PANEL B; SEE S13 Table FOR PRIMER SEQUENCES); RED FONT INDICATES REGIONS DELETED IN 6E CELLS BASED ON PCR ANALYSIS (B). B, PCR products for primer pairs 1–17, indicted in panel A, were analyzed by gel electrophoresis. PCR analysis of genomic DNA isolated from 6E and 9E cells identified large deletions within the viral E1, E2, L2 and L1 genes in 6E cells, including the regions targeted by the 4C viewpoint 1 and 2 non-reading primers. V1-V3, 4C Viewpoints; NTC, no template control. (PDF) [file ppat.1013454.s002.pdf]
