# Supplementary material for: Human papillomavirus genomes associate with active host chromatin during persistent viral infection
Source: PLoS Pathog. 2025 Sep 2;21(9):e1013454. doi: 10.1371/journal.ppat.1013454 (PMC12440168; doi:10.1371/journal.ppat.1013454)
Supplement: Attachment — Submitted filename: Response to Reviewers_FINALv2.pdf [file ppat.1013454.s017.pdf]

Response to Reviewers **(responses in blue)**
We are very grateful for the reviewers' suggestions and appreciation of our manuscript. Their suggestions have substantially improved our manuscript.

Reviewers' Comments

Reviewer #1:

Part I - Summary

Warburton et al perform HiC and 4C analyses on cells with HPV 31 episomes and show that viral genomes often associate with transcriptionally active compartments in open chromatin regions. Using ChiP-seq for Brd4 and H3K27ac the authors report a linkage to regions containing super enhancers. Additional analyses examine published double strand BREAK data sets from normal cells to conclude that the sites of association are often linked to sites of viral genome association. This suggests a potential linkage of viral integration sites to regions susceptible to double strand DNA break formation. This is an interesting and detailed bioinformatic analysis of sites at which HPV genomes associate with host chromatin and potential integration sites. The manuscript is densely written and could benefit from expanded discussion of the points discussed below. In the second part of their study, the authors make comparisons between different cell lines including normal NHEKs and HPV positive which this needs to be described more fully and the implications of using such lines discussed in more detail. Several points should be addressed:

Reviewer #1 commented that the manuscript is "densely written". To address this, we have removed methodological details from the results section when also described in the Methods section and expanded the discussion as suggested.

Part II – Major Issues: Key Experiments Required for Acceptance

1. What is the difference between SM159 and SM163? In Figure 1C there are significant differences in viral-host trans interactions between the two cell lines. What accounts for these differences? Could these interactions be dynamic and change as a function of passage? This needs to be stated more directly in the text as it is only evident when examining the figure. The implication of this variability with different isolates on their conclusions needs to be discussed in detail.
    a. SM159 and SM163 are two biological replicates of HiC analysis in 9E cells. We have updated the names of samples SM159 and SM163 to Replicates 1 and 2, respectively, in Figure 1, Figure S1, Table S1, Table S8 and the associated text to avoid any confusion to the readers.
    b. The higher number of interactions mapped in HiC Replicate 2 may result from experimental variability often observed by one of us (JJM) in biological replicates of HiC experiments. It also possibly reflects differences in the fixation methods used; Replicate 1 and 2 cells were crosslinked either in situ or in suspension, respectively. These details have been added to lines 116-118 of the Results section, in addition to the previous description in the Methods section (lines 439-442).

2. (Figure 8) The ATAC-seq, H3K27ac/Brd4 ChIP-seq, and DSBcapture are all performed on different cell lines (9e, W12, and NHEKs, respectively). Is this the best comparison? These assays are admittedly expensive and time consuming and may be the reason for the choices but particularly the use of NHEKs alone to look at DNA breaks is not ideal. W12 and CIN 612 are more closely aligned. The

reviewer is correct that these assays are expensive and time consuming, and in addition this is not a technique that we have tried or established in our laboratory. As a comparison, we included a profile plot of the three datasets at TSS (-/+ 5Kb) in Figure 8A to show that all three datasets are highly enriched at these regions. This has been emphasized in lines 316-322 of the text. We have also discussed the use of different keratinocyte lines as a limitation of the study in lines 405-409 of the Discussion section. Despite the use of different cell lines, there is a remarkable correlation between the DNA breaks in the original study in NHEK cells, our H3K27ac ChIP peaks in W12 cells, and ATAC seq in 9E cells (see TP63 locus in Figure 8E). We have also carried out further correlation analysis of the BREAK-seq datasets as requested in point #4 below.

3. The authors use a published DSBcapture dataset from NHEKs which are normal cells. The levels of DNA breaks are increased in CIN 612 cells relative to normal cells and the use of DSBcaptuire with this HPV positive line could strengthen the conclusions. Viral-host trans interactions maybe enhanced by the induction of higher levels of DNA breaks induced by viral proteins. As mentioned above, it is not feasible for us to undertake DSBcapture experiments at this time. However, this is an excellent and important point as several studies have shown that HPV infection (and in particular 9E cells) have increased DNA breaks and this could enhance the frequency of integration at these additional sites. We have added a paragraph to the Discussion (lines 406-409) describing this.

4. (Figure 8B-D) Increased DSBcapture reads were observed in the 50kb flanking regions from the called HiC, 4C, and integration breakpoint peaks. Do these increased DSBcapture reads correlate with the called DSBcapture peaks from Lensing el al, 2016? The y-axes of the figure 8B-D is not clearly labeled and this needs to be adjusted (Read counts vs read counts normalized to input, etc).

    a. We have obtained the DSBcapture peaks from the Lensing study and correlated this with our HiC, 4C, and integration breakpoint datasets. This analysis is now described in the manuscript (lines 333-336) and included as a new supplementary table (Table S13).
    b. We have fixed the y-axes in Figure 8B-D as suggested.

Part III – Minor Issues: Editorial and Data Presentation Modifications:

(No Response)

Reviewer #2:

Part I - Summary

The manuscript by Warburton and colleagues use Hi-C and 4-C sequencing of the HPV31 Cervical keratinocyte line CIN612-9E to identify host chromosome association with a variety of other genomic methods. They conclude that viral genomes associate with active transcription regions. While they claim that this serves to maintain active viral gene expression, this was not tested. A limitation of the study is the use of genomic datasets from other cell lines instead of the 9E line.

Part II – Major Issues: Key Experiments Required for Acceptance

The major issue in this manuscript is the co-mingling of so many diverse datasets from a variety of cell lines. While this might not be an issue if every line tested behaves similarly whether it has HPV16 or HPV31, if the viral genome is integrated or episomal. Are the super enhancers identified by ChIP-seq of HPV16 keratinocytes going to be the same in the 9E cells? It certainly is possible but this issue should be addressed. Similarly, are the DSBs identified in NHEK cells relevant to the 9E cells. Again, they might be relevant but the question was not addressed. This assessment/critique is similar to that of Reviewer #1 and our response is similar and described above.

Part III – Minor Issues: Editorial and Data Presentation Modifications

1. Fig2C. It seems odd that all the correlations among the viewpoints are between 0.61 and 0.64. Was there any biases in the calculations? There were no biases in the calculations. We have checked the original data and decided to display the heatmap in Figure 2C using a Pearson correlation coefficient to account for the height of the averaged bedgraph files for each viewpoint, with a complete range from 0-100 for better visualization. The correlations among the three viewpoints are now between 0.36 and 0.48, which indicates that none of the datasets are considered an outlier.

2. Line 180-181. Why do you assume that all of the 4C-seq interactions with other chromosomes are background? Is it possible that the integrated viral genome could connect with other transcriptional active regions on other chromosomes? We did not mean to imply that all 4C interactions in 6E cells were background and certainly there will be interactions with other chromosomes. We have clarified this point in lines 180-184 of the Results section and deleted text in lines 357-359 of the figure legend that would imply as such.

3. Line 206 why not illustrate the connections between HPV and MYC, E2F2, and CASP8 similar to Figure 3? As suggested, we have illustrated the 9E 4C averaged bedgraph data as a circo plot and genome alignments at chromosomes 1, 2 and 8 at the E2F2, CASP8 and MYC gene loci, respectively, in Figure 4C. We also updated Figure 3 to include the scale of the circo plot for the 6E 4C averaged bedgraph data for comparison to the 9E data.

4. Line 244-246. Fig 5G. How was this region of the genome selected for illustration? Are there any relevant genes in this region? Myc? E2F2? CASP8? The region was selected based on it having the highest ATAC-seq signal in the HiC dataset, and two of the top five highest signals in the 4C dataset. We have updated Figure 5G to include the genes spanning this region that are expressed in cervical keratinocytes and noted those of biological relevance in the associated text in lines 250-257.

5. Line252-255. Figure 6, how sure are you that the super enhancers from a different cell line are relevant? Super-enhancers are highly tissue-specific, and we have previously shown that >80% of ChIP-seq peaks for the super-enhancer markers Brd4/H3K27ac that we mapped in HPV16-positive W12 cells overlapped with ENCODE defined enhancers in NHEK cells. We therefore feel confident that these regulatory elements are highly conserved between W12 and 9E cervical keratinocytes. To address the reviewer's comment, we have discussed the similarities between W12 and NHEK enhancers in lines 262-267 of the results section.
6. Figure 7. Is there any specific information about integration of HPV31 in cervical cancer or does every high risk genotype have similar hotspots? From the literature, HPV31-positive cervical tumors

represent <10% of tumors with integrated viral genomes, and integrated HPV31 genomes from several cervical tumors have been shown to overlap integration hotspots. Furthermore, the genomes of several HPV types (HPV16, HPV18, HPV45 and HPV59) were found integrated at integration hotspots in multiple tumor samples (17, 72, 73), indicating that integration into these regions is common among different high-risk HPV types. We have discussed this in lines 413-420 of the manuscript.

Reviewer #3:

Part I - Summary

In this manuscript, Warburton et al. use chromosome conformation capture assays (Hi-C and 4C-Seq) to map the association of the HPV31 genome—either in its extrachromosomal or integrated form—with host chromatin. Their findings regarding the integrated form of the viral genome are consistent with previous studies based on curated dataset analyses or cell lines containing integrated HPV genomes (Warburton, Genomic Medicine, 2021; Singh, Molecular Oncology, 2023), showing that the viral genome is associated with active chromatin regions without a specific chromosome preference.

An important finding of this study is the association of the extrachromosomal genome with transcriptionally active regions (as shown by ATAC-Seq experiments and previously published datasets for Brd4 and H3K27Ac ChIP-seq), HPV integration hotspots (from previously published HPV integration datasets), and regions prone to double-strand breaks (from DSBCapture datasets). While the authors report that the association of the extrachromosomal viral genome is not limited to a specific region of the viral genome, the intersection of Hi-C and 4C-Seq data identified 19 regions of the human genome—including MYC, E2F2, and CASP8—that are common across analyses. These data support a model in which the extrachromosomal HPV genome is enriched at specific genomic locations. These findings are significant as they reveal a novel correlation between the localization of the extrachromosomal form of HPV and integration sites. Minor changes are discussed below.

Part II – Major Issues: Key Experiments Required for Acceptance

Reviewer #3: No major issue has been found.

Part III – Minor Issues: Editorial and Data Presentation Modifications

1. Figure 1 presents data on the association of extrachromosomal HPV with human chromosomes using Hi-C in CIN912 9E. Since the authors later show that HPV31 is integrated into chromosome 4 in 6E, could the authors comment on the absence of association with this analysis (1D)? Additionally, could the authors explain the lower number of associations of the HPV genome with the human genome observed in SM159 compared to SM163 in this figure?
    a. Notably, we did not observe association of extrachromosomal HPV genomes with the same site as the integration locus in 6E cells. However, integration and clonal selection of cells with integrated genomes is a stochastic and rare event. This point has been discussed in lines 411-413 of the Discussion
    b. This comment is similar to that of Reviewer #1 and was addressed above

2. The results obtained using Hi-C to map the regions of association on the HPV genome are somewhat confusing. As the authors mention the limited reliability of this technique for such analysis, these

data might be better suited for inclusion in a supplementary figure, allowing the main focus to remain on the 4C-Seq analysis. As mentioned above, we have renamed the HiC samples to Replicates 1 & 2 to better clarify the HiC datasets. To allow us to better compare the HiC and 4C datasets, we would prefer to retain Figure 1 in the main manuscript.

3. Since the interaction between Brd4 and E2 is required for tethering the HPV genome to daughter cells, the observed interaction of the extrachromosomal viral genome with Brd4- enriched regions is expected. Could the authors comment on any potential bias introduced by this tethering in their analyses? For instance, were most cells in the same phase of the cell cycle during the Hi-C and 4C-Seq experiments? In a previous study from our laboratory (Jang, 2014), we mapped genomic binding sites for Brd4 and HPV E2 in cervical keratinocytes. We named these regions as PEB-BLOCS or Persistent E2 and BRD4-Broad Localized Enrichments of Chromatin. These loci were termed "persistent" because they persisted throughout the cell cycle, including during mitosis when many factors are displaced from chromatin. They are also broad, transcriptionally active chromatin domains and so we think that this correlated well with the current data, as discussed in lines 371-384 of the Discussion. For both techniques (HiC and 4C), the cells were cultured using similar methods and harvested when ~60% confluent. Thus, they likely represent similar populations of cell cycle distribution.
