# Supplementary material for: Human papillomavirus genomes associate with active host chromatin during persistent viral infection
Source: PLoS Pathog. 2025 Sep 2;21(9):e1013454. doi: 10.1371/journal.ppat.1013454 (PMC12440168; doi:10.1371/journal.ppat.1013454)
Supplement: Attachment — Submitted filename: Response to Reviewers_08-08-2025.docx [file ppat.1013454.s018.docx]

**Response to Reviewers** (responses in blue)

We are very grateful for the reviewers’ suggestions and appreciation of our manuscript. We have addressed the minor comment raised by Reviewer 1.

**Reviewers’ Comments**

**Reviewer #1:**

**Part I - Summary**

In the revised version of this manuscript, the authors have addressed and clarified many of the points raised in the initial review. The manuscript provides important insights regarding interactions between HPV genomes and host chromosomal sites through association with open chromatin, super enhancers and DNA breaks.

**Part II – Major Issues: Key Experiments Required for Acceptance**

No major issues

**Part III – Minor Issues: Editorial and Data Presentation Modifications**
In response to questions about differences in HPV 31 trans-interactions with various host chromosome bins (Figure 1) between Replicate 1 and Replicate 2, the authors suggest this could be due to differences in fixation methods. Equally plausible is the possibility that these interactions are dynamic and, aside from associations with super enhancers, these interactions could change with cell cycle, passage or cell clone. It might be useful to mention this possibility.

We have added the following text on p5.

A higher number of total and *trans*-interactions were mapped in HiC Replicate 2, compared to Replicate 1. It has been shown that chromosome topology is very dynamic and can change considerably during the cell cycle [22, 23] and even after continued passage of cells [24]. In our case, care was taken to use similar early cell passes for each replicate, and to harvest cells at similar confluency (which should affect cell cycle distribution). However, the difference in the number of interactions could have been influenced by the fixation conditions used; Replicate 1 and 2 cells were crosslinked in situ or in suspension, respectively.

**Reviewer #2:**

**Part I - Summary**

The revised manuscript from Warburton and colleagues demonstrate that HPV31 genomes preferentially associate with transcriptionally active A compartments of host chromatin, regions of open chromatin defined by ATAC-seq, super-enhancers defined by Brd4 and H3K27ac ChIP-seq and double-stranded DNA breaks associated with transcriptionally active sites. These association sites viral genome association sites were highly correlated with genomic loci previously identified as common HPV integration sites in cervical cancers.

**Part II – Major Issues: Key Experiments Required for Acceptance**

No new experiments required

**Part III – Minor Issues: Editorial and Data Presentation Modifications**
Revised manuscript has Mae several changes to clarify the use of divergent genomic databases

**Reviewer #3:**

**Part I - Summary**

Although the analyses in this study remain based on data from different cell lines, the authors now acknowledge this limitation and provide clearer context for readers to better assess the findings. The authors addressed most of the minor concerns raised by the reviewers, improving the manuscript’s readability.

**Part II – Major Issues: Key Experiments Required for Acceptance**

(No Response)

**Part III – Minor Issues: Editorial and Data Presentation Modifications**
(No Response)
